# Updating ‘Perceptions and opinions on the COVID-19 pandemic in Flanders, Belgium’ with data of two additional waves of a longitudinal study^✩^

**DOI:** 10.1016/j.dib.2022.108010

**Published:** 2022-03-06

**Authors:** David De Coninck, Leen d'Haenens, Geert Molenberghs, Anja Declercq, Christophe Delecluse, Evelien Van Roie, Koen Matthijs

**Affiliations:** a Centre for Sociological Research, KU Leuven, Belgium; b Institute for Media Studies, KU Leuven, Belgium; c Interuniversity Institute for Biostatistics and Statistical Bioinformatics, Data Science Institute, Hasselt University, Belgium; d Interuniversity Institute for Biostatistics and Statistical Bioinformatics, KU Leuven, Belgium; e LUCAS, KU Leuven, Belgium; f Department of Movement Sciences, KU Leuven, Belgium

**Keywords:** COVID-19, Coronavirus, Belgium, Longitudinal, Attitudes, Public health, Pandemic, Crisis

## Abstract

Adding to longitudinal data of three waves that were presented in an original dataset on perceptions and behaviours regarding government measures, fear of getting ill, and media use during the COVID-19 pandemic in Flanders (Belgium), this article presents information on two additional waves that were collected at two key moments in the pandemic in the same region: in late August 2020 (W4; as infection rates increased again; N = 505) and in the middle of March 2021, exactly one year after the first data collection (W5; N = 408). In W4 and W5, new respondents were added to the longitudinal sample to strengthen cross-sectional analyses. Additional information on informal care and physical activity was also collected. These data may be of interest to researchers who wish to explore dynamics of fear and attitudes towards public health measures during this particularly challenging time.

## Specifications Table

**Table:** 

Subject	Public Health and Health Policy; Social Sciences
Specific subject area	Attitudes towards public health; Fear of disease; Media consumption; Informal care; Physical activities
Type of data	Table
How the data were acquired	Five-wave online survey among the adult population in Flanders, Belgium
Data format	Raw, analysed, and filtered.
Description of data collection	We collected the data in cooperation with a Belgian survey agency and selected the methodology for its cost-effectiveness in large-scale and longitudinal research. Respondents received an e-mail asking them to participate in a survey without specifying the subject matter. Following an initial three rounds, data were collected at two additional key moments in the pandemic among adults in Flanders, Belgium: late August 2020 (as infections increased again; N = 1,000), and middle of March 2021 (one year following the first measurement; N = 1,646). The initial response rate was about 35%, the drop-out rate between Wave 1 and Wave 5 was 59%.
Data source location	Institution: KU Leuven City/Town/Region: Leuven Country: Belgium
Data accessibility	Repository name: Perceptions and opinions on the COVID-19 pandemic in Flanders, Belgium: Data from a five-wave longitudinal study Data identification number: http://dx.doi.org/10.17632/mhx3p7w3d6.9 Direct URL to data: https://data.mendeley.com/datasets/mhx3p7w3d6/9
Related data article	D. De Coninck, L. d’Haenens, K. Matthijs, Perceptions and opinions on the COVID-19 pandemic in Flanders, Belgium: data from a three-wave longitudinal study, Data in Brief 32 (2020) 106060, doi:10.1016/j.dib.2020.106060.

## Value of the Data

• These data may be used to analyse quarantine strategies and contexts for their social acceptability, understand the consequences of the epidemic on informal care and physical activity, determining best ways to apply knowledge about infection prevention and control, and enhance (or develop) an ethical framework for outbreak response.
• The data presented fill an important gap: there is currently very little longitudinal COVID-19 research that combines indicators on fear and attitudes with measures on news media consumption. This allows for new insights in a field which is rapidly evolving, and where researchers and policy makers alike are looking for new insights. There are key advantages to this design: even when selection is somewhat biased, it is likely that within-person evolution is estimated with sufficient accuracy.

## 1 Data Description

The data presented in this article were collected during the COVID-19 pandemic in Flanders, Belgium and build on an earlier dataset [1,2]. In addition to the three waves that were included in the first dataset, data were also collected two more times; once in 2020 and once in 2021: from August 18 to August 31, 2020 (as infection rates had started to increase again; N = 1,000), and from March 17 to April 5, 2021 (as a third wave of infection rates hit Belgium, exactly one year after the first; N = 1,646).

In Wave 4 and Wave 5, we maintained the original longitudinal design but also added new respondents to strengthen potential cross-sectional analyses on the data of these individual waves. More specifically, 505 respondents in Wave 4 had participated in all previous waves. This was then supplemented with new respondents who had not participated in the study before to reach N = 1,000. Wave 5 contained 408 respondents who had participated in all waves of the study, and this was supplemented with new respondents to reach N = 1,646 Table 1. shows the distribution of respondents who participated in these waves by several socio-demographic characteristics and per wave, while Table 2 presents mean scores of selected indicators on perceived vulnerability to disease, attitudes towards public health measures, news media consumption, resilience, physicial activities and informal care indicators.

**Table 1:** Socio-demographic characteristics of participants in wave 4 and 5 (in %, unless otherwise specified).

	Wave 4 (N = 505)	Wave 5 (N = 408)
**Gender**		
Male	52.9	57.6
Female	47.1	42.4
**Mean age** (in years)	49.31	46.41
**Educational attainment**		
No degree/Primary degree	1.8	3.1
Secondary degree	54.7	63.7
Tertiary degree	42.5	33.2
**Perceived income** (average score)^a^	4.02	3.93
**Political ideology** (average score)^b^	4.30	4.31
**Living environment**		
Large city	9.1	7.6
Suburbs	17.0	16.6
Small city	22.9	22.8
Village	44.8	48.3
Countryside	5.9	4.7
Other	0.2	0
**(Work) situation**		
Full-time job	47.5	55.2
Part-time job	12.2	10.8
Temporarily disabled	1.8	1.5
Permanently disabled	3.6	4.2
Student	3.2	5.5
Houseman/housewife	3.8	4.6
Unemployed	2.7	3.8
Retired	27.5	15.2
**Respondent was requested to work from home during COVID-19 crisis**		
Yes	32.3	41.3
No	32.4	28.1
*N/A*^c^	*35.3*	30.5
**Company that respondent worked at closed down due to COVID-19 crisis**		
Yes	17.8	21.6
No	45.7	49.8
*N/A*^c^	*36.4*	*28.6*
**Informal care indicators**^d^		
Informal carer (yes)	8.5	15.7
***Time spent on care since COVID-19 crisis***		
More time	38.0	37.5
Less time	6.9	8.2
Same time	55.1	54.3
***Perceived burden of care since COVID-crisis***		
More difficult	40.9	36.5
Easier	2.8	2.2
The same	56.3	61.3

Note:

a Perceived income was measured by asking respondents about easily they make do with their current income, ranging from 1 = very difficult to 5 = very easily.

b Political ideology was measured through the following question: ‘When it comes to politics, people talk about `left' and `right'. Where would you place yourself on the scale below, where 1 stands for far left and 7 for far right?’

c Denotes respondents that did not work prior to crisis (disabled people, students, housemen/wives, unemployed people, retirees).

d Due to a low number of informal carers in the longitudinal sample, the full samples of W4 (N = 1,000) and W5 (N = 1,646) were used for these descriptives.

**Table 2:** Mean scores of selected indicators on perceived vulnerability to disease, news media consumption and attitudes towards public health measures, resilience, physical activity, and informal care.

	Wave 4 (N = 505)	Wave 5 (N = 408)
**Perceived vulnerability to disease**		
It really bothers me when people sneeze without covering their mouths.	6.01	5.89
If an illness is ‘going around’, I will get it.	2.77	3.07
I am comfortable sharing a water bottle with a friend.	2.95	2.93
I do not like to write with a pencil someone else has obviously chewed on.	5.33	5.32
My past experiences make me believe I am not likely to get sick even when my friends are sick.	3.70	3.66
I have a history of susceptibility to infectious disease.	2.43	2.66
I prefer to wash my hands pretty soon after shaking someone's hand.	4.66	4.31
In general, I am very susceptible to colds, flu and other infectious diseases.	3.18	3.30
I dislike wearing used clothes because you do not know what the last person who wore it was like.	4.53	4.53
I am more likely than the people around me to catch an infectious disease.	2.74	2.87
My hands do not feel dirty after touching money.	4.09	4.15
I am unlikely to catch a cold, flu or other illness, even if it is ‘going around’.	3.56	3.45
It does not make me anxious to be around sick people.	3.71	3.93
My immune system protects me from most illnesses that other people get.	3.83	4.01
I avoid using public telephones because of the risk that I may catch something from the previous user.	3.45	3.21
**News media consumption**		
Public television	3.29	3.05
Commercial television	2.64	2.65
Public radio	2.95	2.85
Commercial radio	2.14	2.19
Quality newspaper	2.08	1.99
Popular newspaper	3.01	2.81
Social media of public news/quality newspaper	2.33	2.13
Social media of commercial news/popular newspaper	2.43	2.17
**Attitudes towards public health measures**		
Measures are necessary to protect population	3.88	3.96
Fear of economic crisis	4.20	4.25
Fear of loneliness	2.39	2.66
Quarantine when feeling sick	3.79	3.80
Government handles crisis well	2.69	2.72
**Brief Resilience Scale**		
I tend to bounce back quickly after hard times	3.72	3.65
I have a hard time making it through stressful events.	2.58	2.74
It does not take me long to recover from a stressful event.	3.34	3.24
It is hard for me to snap back when something bad happens.	2.92	3.02
I usually come through difficult times with little trouble	3.38	3.31
I tend to take a long time to get over setbacks in my life.	2.64	2.76
**Physical activities**^a^**^,^**^b^		
Vigorous physical activity in last 7 days	-	2.50
Vigorous physical activity prior to crisis	-	2.74
Moderate physical activity in last 7 days	-	3.21
Moderate physical activity prior to crisis	-	3.52
Walking in last 7 days	-	4.43
Walking prior to crisis	-	4.16
Sitting in last 7 days (in hours)	-	7.03
Sitting prior to crisis (in hours)	-	6.30
**Informal care**^b^		
Satisfaction collaboration with other informal carers – pre-COVID-crisis	3.24	4.09
Satisfaction collaboration with other informal carers – during COVID-crisis	3.27	4.13
Perceived burden of informal care	5.47	5.29
Satisfaction with professional care	4.34	4.49
Satisfaction collaboration with professional carers	4.20	4.41

Note:

a Data presented here are mean scores, but the dataset also contains information on duration of these activities (in hours and minutes).

b The full samples of W4 (N = 1,000) and W5 (N = 1,646) were used for these descriptives.

## 2 Experimental Design, Materials and Methods

Although several population surveys were conducted into attitudes regarding COVID-19 or public health measures, few were longitudinal. An inherent shortcoming of cross-sectional surveys is that they cannot make any causal claims, while longitudinal studies provide more context regarding the complex nature of such relationships. To recapitulate, we developed an online public opinion survey, to be carried out amongst a sample of Flemish residents aged 18 to 70, representative for gender, age, educational attainment, and province. This survey was distributed at three key moments during the COVID-19 pandemic in Flanders, Belgium. The survey was distributed by iVOX, a Belgian market research and online polling agency. Upon completion of the survey, respondents were given several virtual points. After collecting a certain number of points, they were able to purchase gift coupons for restaurant visits, trips, and other activities, from the website of the survey agency. The survey consisted of eight themes: socio-demographic characteristics, perceived vulnerability to disease, media- and news consumption, contact with the coronavirus, attitudes towards public health measures, and personality characteristics, perceived realistic and symbolic COVID-19 threat, informal care burden (W4 and W5 only), vaccine hesitancy, and changes in physical activities (W5 only). In what follows, we highlighted several measures that were added in the new dataset. All data were processed and cleaned through SPSS.

### 2.1 Informal care

Through a series of questions added in W4 and W5, we assessed whether respondents were informal carers (yes/no), whom their main person with care needs was, and whether they found it difficult to provide this informal care at the moment of the study (1 = not at all difficult, 11 = extremely difficult), and whether this has changed (1 = more difficult since COVID-19, 2 = easier since COVID-19, 3 = the same). Further, we also assessed their time spent on informal care (1 = more since COVID-19, 2 = less since COVID-19, 3 = the same), whether they collaborated with other informal carers and how satisfied they were with this collaboration (1 = very dissatisfied, 5 = very satisfied), whether their main person with care needs received professional care, how satisfied they were with this care and the collaboration with these carers.

### 2.2 Physical activity

Using the International Physical Activity Questionnaire – short form [3], we measured whether respondents’ levels of physical activity (PA) and sedentary behaviour (SB) changed from before the pandemic. More specific, this questionnaire asked to what extent, i.e. frequency (in days, 1 = never; 8 = every day) and duration (in minutes per day), respondents performed three specific types of activity for at least 10 minutes both before the pandemic and during the past seven days: 1) vigorous PA ((i.e. activities much harder than normal), 2) moderate PA (i.e. activities somewhat harder than normal) and 3) walking. In addition, the daily amount of sitting (in hours and minutes per day) was recorded.

## Ethics Statements

Ethical review and approval was not required for the study on human participants in accordance with the local legislation and institutional requirements. All data was collected in accordance with GDPR requirements. Informed consent was obtained from respondents prior to completion of the online survey.

## CRediT Author Statement

**David De Coninck:** Conceptualization, Methodology, Software, Writing – original draft preparation; **Leen d'Haenens:** Conceptualization, Methodology, Writing – review & editing, Supervision; **Geert Molenberghs:** Methodology, Writing – review & editing, Supervision; **Anja Declercq:** Methodology, Writing – review & editing, Supervision; **Christophe Delecluse:** Methodology, Writing – review & editing, Supervision; **Evelien Van Roie:** Methodology, Writing – review & editing; **Koen Matthijs:** Conceptualization, Methodology, Writing – review & editing, Supervision.

## Declaration of Competing Interest

The authors declare that they have no known competing financial interests or personal relationships that could have appeared to influence the work reported in this paper.
